# Emerging Role and Potential Therapeutic Application of TRIM Proteins in Cardiovascular Diseases

**DOI:** 10.3390/biom16050676

**Published:** 2026-05-02

**Authors:** Yiyang Cui, Yuxuan Wen, Xinling Wang, Yu Xu, Meixiu Jiang

**Affiliations:** 1 The Queen Mary School, Jiangxi Medical College, Nanchang University, 999 Xuefu Road, Nanchang 330031, China; jp4217122151@qmul.ac.uk (Y.C.); jp4217122152@qmul.ac.uk (Y.W.); 2 Jiangxi Province Key Laboratory of Bioengineering Drugs, The National Engineering Research Center for Bioengineering Drugs and the Technologies, Institute of Translational Medicine, Jiangxi Medical College, Nanchang University, 999 Xuefu Road, Nanchang 330031, China; 407400250014@email.ncu.edu.cn; 3 School of Pharmacy, Jiangxi Medical College, Nanchang University, 999 Xuefu Road, Nanchang 330031, China; 406700250023@email.ncu.edu.cn

**Keywords:** TRIM proteins, E3 ubiquitin ligase activities, cardiovascular diseases, mechanisms, clinical perspective

## Abstract

Cardiovascular diseases have high mortality rates and present a high burden on society and the global healthcare system. A large quantity of drugs have been developed, such as aspirin, ACE inhibitors, beta-blockers, and statins. Although these traditional drugs have decreased the morbidity and mortality of cardiovascular diseases, they still have multiple limitations. Due to their shortcomings, researchers have continued to search for novel targets for drug treatment. The tripartite motif (TRIM) protein family is a superfamily with E3 ubiquitin ligase activity and involves diversified processes including proliferation, development, signal transduction, and immune regulation. The latest research has shown that TRIM proteins participate in the progression of cardiovascular diseases, such as cardiac hypertrophy, cardiac ischemia–reperfusion injury, heart failure, hypertension, atherosclerosis, and so on. In this review, we summarize the structure and function of TRIM proteins, as well as the mechanisms of their involvement in various cardiovascular diseases, aiming to raise awareness of the importance of TRIM proteins in cardiovascular disease research and treatment. Advancing our understanding of mechanisms mediated by TRIM proteins may emphasize their contributions to cardiovascular diseases and provide the opportunity to develop novel and targeted therapeutic strategies to combat cardiovascular diseases.

## 1. Introduction

Cardiovascular diseases (CVDs) remain the paramount cause of global mortality, with approximately 17.9 million deaths each year, which represent a persistent and formidable challenge to public health systems worldwide [1]. Demographic aging and the growing prevalence of modifiable risk factors, including obesity, tobacco smoking, physical inactivity, diabetes, elevated blood cholesterol and high blood pressure, further exacerbate this challenge [2]. Notwithstanding substantial progress in therapeutic strategies over decades ranging from pharmacotherapies to interventional procedures and device-based therapies, these advancements have not overcome the fundamental challenges in the clinical management of CVDs.

The contemporary clinical dilemma for CVDs is twofold: Firstly, current medications, such as statins, antihypertensives, and antiplatelet drugs, offer predominantly symptomatic relief and can modestly delay disease advancement. Critically, they are largely ineffective at arresting or regressing the fundamental pathological remodeling of cardiac and vascular tissues. Furthermore, their utility is often tempered by drug-related side effects. Secondly, the pathogenesis of CVDs is highly heterogeneous, resulting in widely variable treatment responses among patients [3,4,5,6,7]. Consequently, there is an urgent need for novel interventions that are more precise and tailored to underlying mechanisms.

The current research on the mechanisms of cardiovascular diseases mainly focuses on several interrelated aspects: chronic inflammation, oxidative stress, impaired autophagy, mitochondrial dysfunction, regulated cell death (e.g., pyroptosis, apoptosis, ferroptosis), metabolic disorders, and epigenetic modifications, as well as vascular endothelial dysfunction [8,9,10,11,12,13,14]. However, the precise upstream regulatory mechanisms for these complex processes remain largely unknown. In recent years, post-translational modifications (PTMs) have gained prominence for their pivotal role in deciphering the intricate pathogenesis of cardiovascular diseases, owing to their dynamic reversibility. Through novel modifications such as phosphorylation, acetylation, ubiquitination, and succinylation, PTMs precisely regulate protein functions and are widely involved in the core signaling pathways of CVDs [13,15].

The TRIM protein family has more than 80 members, characterized by the RBCC structure and variable C-terminal domains with a high degree of structural diversity, which determines the specificity of function of each protein. It has been proven that TRIM family proteins have the E3 ubiquitin ligase activity, which involves the ubiquitylation of proteins, except for TRIM44, which functions as a deubiquitinase [16]. Besides TRIM proteins playing an important role in many biological processes, including proliferation, differentiation, transcription, apoptosis, and regulating the immune responses, TRIM family proteins are also involved in many kinds of diseases, including cancer, immunological diseases, and developmental disorders [17]. Among regulators of PTMs, TRIM family proteins have attracted much attention due to their crucial role in CVDs in recent years, primarily by mediating substrate ubiquitination through their E3 ligase activity [18].

Therefore, this review introduces the TRIM family and summarizes the multifaceted roles of TRIM proteins in CVDs, including cardiac hypertrophy, acute myocardial ischemia–reperfusion injury, hypertension, arrhythmias, and heart failure. It emphasizes their contribution to pathological pathways and their potential as therapeutic targets. Deepening our understanding of the mechanisms mediated by TRIM proteins may provide the opportunity for innovations and precise strategies in the treatment of cardiovascular diseases.

## 2. TRIM Family

### 2.1. Structures of TRIM Family Proteins

**Figure 1:**
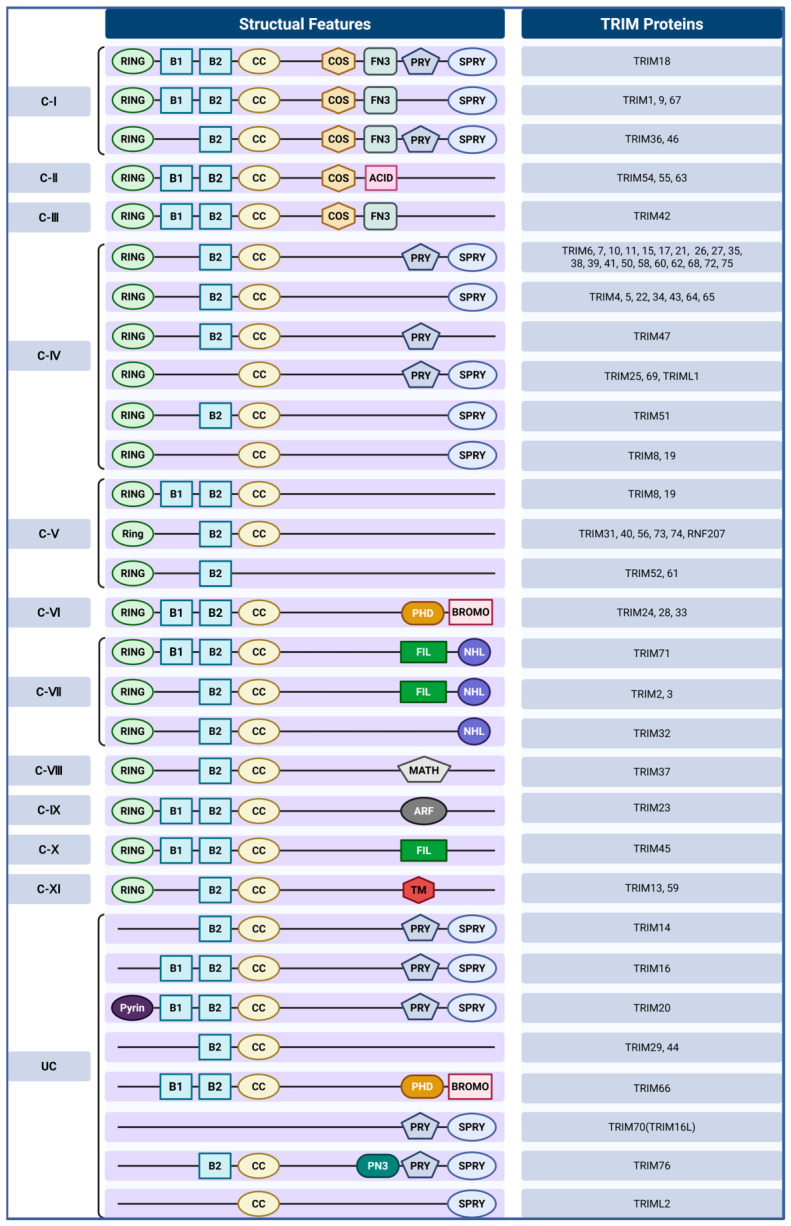
**Structural classification of the human TRIM family.** The TRIM family is categorized based on the C-terminal domain into 11 canonical subfamilies (C-I to C-XI) and an unclassified (UC) group. The canonical subfamilies are defined by a conserved N-terminal RBCC motif, which consists of a RING domain, one or two B-box domains (B1 and B2), and a coiled-coil (CC) region. The diversity in the C-terminal domains following this motif, such as PRY-SPRY, PHD-BROMO, and FIL, dictates the functional specificity of each subfamily. The unclassified (UC) group comprises members with varied, non-canonical domain arrangements, including several that lack the characteristic N-terminal RING domain. Representative members for each structural class are listed.

The TRIM protein family contains more than 80 members in humans, each defined by a conserved N-terminal RBCC motif coupled to a highly variable C-terminal domain. The RBCC motif comprises a RING finger domain, one or two B-box domains, and a coiled-coil region. The RING finger domain provides the catalytic platform for interaction with E2–ubiquitin conjugates and mediates ubiquitin transfer to substrate proteins, thereby conferring the intrinsic E3 ubiquitin ligase activity of TRIMs [19,20,21,22]. Following the RING domain, the B-box domain mediates protein–protein interactions, contributes to higher-order polymer formation, and influences the substrate recognition specificity [21,23]. The coiled-coil domain, positioned after the B-box, contributes to homo- or heterodimerization of TRIM proteins, promotes the assembly of higher-order oligomers and functional macromolecular complexes, and participates in the subcellular localization and distribution of substrate proteins (Figure 1) [22].

The C-terminal domain, by contrast, exhibits considerable diversity, determining substrate interaction specificity and serving as the key feature underlying functional classification into eleven subfamilies (C-I to C-XI), along with an unclassified group encompassing members lacking a canonical RING domain (Figure 1) [24]. The PRY/SPRY domain (also known as B30.2) is widespread, mediating specific recognition of protein targets and regulating innate immune responses [17,25,26]. The PHD–BROMO tandem module interacts with modified histones to regulate transcription and chromatin remodeling [24,27]. Additional C-terminal modules include the COS domain, involved in dimerization and microtubule binding, the FN3 domain, which interacts with DNA [24,28], and the MATH domain, which binds TNF receptor family proteins and influences transcription factor function [29]. Other domains, such as FIL, NHL, ARF, ACID, and TM, further diversify TRIM protein interactions, subcellular localizations, and signaling capacities [23,30]. Intriguingly, RING-less TRIMs, such as TRIM16, can still exert E3 ligase activity via atypical mechanisms, broadening the functional repertoire of the family [31].

### 2.2. Ubiquitination and Functional Basis of TRIM Proteins

The TRIM family exerts its biological functions mainly as E3 ubiquitin ligases, which play a crucial role in catalyzing protein post-translational modification via ubiquitination [32,33,34]. This process is an ATP-dependent enzymatic cascade: a ubiquitin-activating enzyme (E1) activates and transfers ubiquitin to a ubiquitin-conjugating enzyme (E2). Subsequently, the TRIM protein, functioning as the E3 ligase, must recognize and bind both a specific E2 and the substrate protein, facilitating the transfer of ubiquitin from the E2 to the substrate through its RING domain [21,32,33,34,35,36]. Ubiquitin, a 76–amino acid polypeptide, contains seven internal lysine sites (K6, K11, K27, K29, K33, K48, K63) and an N-terminal methionine (M1). These sites enable polyubiquitin chain formation by linking to the C-terminal glycine (G76) of another ubiquitin [37,38]. The specific topology of these chains forms a complex “ubiquitin code” that dictates distinct functional outcomes.

**Figure 2:**
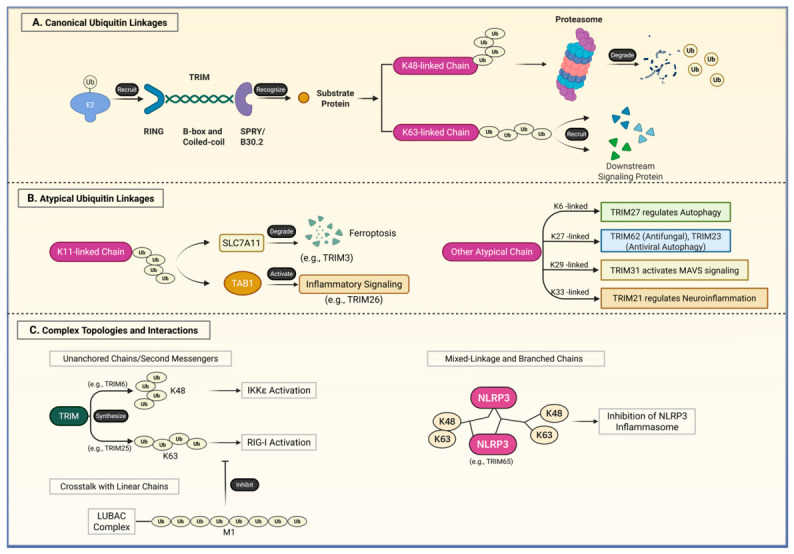
**The diverse mechanisms of TRIM as E3 ubiquitin ligase activity.** TRIM proteins mediate various ubiquitination events with distinct outcomes. (**A**) Canonical Linkages: K48-linked chains target proteins for proteasomal degradation, while K63-linked chains serve as scaffolds for signaling. (**B**) Atypical Linkages: TRIMs utilize non-canonical chains for specialized functions, such as the context-dependent roles of K11 chains in degradation (e.g., TRIM3) or activation (e.g., TRIM26), and the specific signaling roles of K6, K27, K29 and K33 chains. (**C**) Complex Topologies: TRIMs also generate complex signals, including unanchored chains that act as second messengers (e.g., TRIM25, TRIM6), mixed-linkage chains for integrated regulation (e.g., TRIM65), and engage in crosstalk with other pathways like the LUBAC complex.

As “writers” of this code, TRIM proteins mediate the formation of various chain types (Figure 2). Among these, K48 and K63 linkages represent the canonical forms most prevalent in mammals [39]. TRIM proteins frequently utilize K48-linked chains to tag substrate proteins for degradation by the 26S proteasome, a fundamental function in protein quality control [20,24]. In contrast, K63-linked chains predominantly serve as non-degradative signaling scaffolds, mediating processes such as protein transport, protein–protein interactions, and the DNA damage response [20,24]. The versatility of TRIM proteins extends far beyond these canonical linkages, enabling them to regulate highly specific cellular events through atypical and complex ubiquitin topologies. TRIM proteins can utilize atypical linkages (e.g., K11, K6, K27, K33) to regulate ferroptosis, autophagy, antiviral responses, and neuroinflammation in specific contexts [40,41,42,43,44]. Furthermore, some TRIMs exhibit more complex activities. A well-known example is TRIM25, which generates unanchored K63 chains. These chains serve as soluble second messengers to activate RIG-I [45]. This remarkable versatility enables TRIM proteins to integrate protein quality control with the dynamic regulation of intracellular signaling networks, establishing their pivotal role in various biological processes and diseases [46,47]. Consequently, this diverse E3 ligase activity constitutes the fundamental basis of TRIM family influence on cardiovascular pathophysiology.

## 3. TRIM Family Proteins in Cardiovascular Diseases

### 3.1. TRIM Proteins in Cardiac Hypertrophy

**Figure 3:**
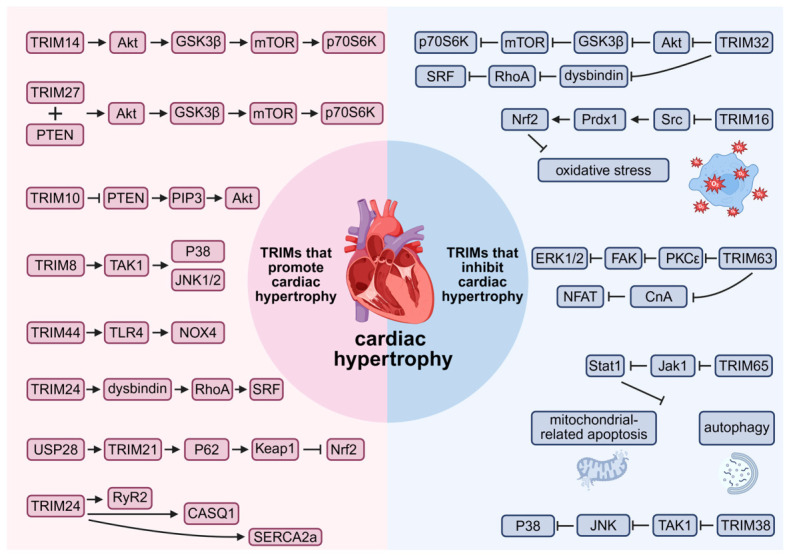
**The role and mechanisms of TRIM proteins in cardiac hypertrophy.** TRIM proteins regulate cardiac hypertrophy through multiple signaling cascades, either promoting (**left panel**) or inhibiting (**right panel**) the pathological process. TRIM14, TRIM27, TRIM10, TRIM8, TRIM44, TRIM24 and TRIM21 promote cardiac hypertrophy via Akt/GSK3β/mTOR, PTEN/PIP3/Akt, MAPK, TLR4/NOX4, RhoA/SRF, calcium handling pathways and P62/Keap1/Nrf2. Conversely, TRIM32, TRIM16, TRIM63, TRIM65, and TRIM38 inhibit cardiac hypertrophy through suppression of Akt/GSK3β/mTOR, RhoA/SRF, oxidative stress, PKCε/FAK/ERK1/2, CnA-NFAT, JAK1/Stat1, and TAK1/JNK/p38 signaling pathways.

Pathological cardiac hypertrophy is a significant risk factor for heart failure and sudden cardiac death [48,49,50]. This pathological process is triggered by various stimulus, including mechanical overload, calcium dyshomeostasis, oxidative stress, and inflammation. These factors activate specific signaling cascades that drive both cardiomyocyte hypertrophy (manifested by cellular enlargement, protein phenotype changes, and contractile alterations) and extracellular matrix fibrosis [51]. Studies have shown that TRIM family proteins participate in regulating signaling pathways and protein turnover related to cardiac hypertrophy by mediating the ubiquitination and degradation of specific substrates (Figure 3), offering new potential directions for the development of future therapeutic strategies.

#### 3.1.1. TRIMs Promote Cardiac Hypertrophy

Over decades, relevant studies have established a complex signaling network, in which the TRIM family proteins regulate cardiac hypertrophy caused by stress within this network [51]. Hou H et al. found that cardiac overexpression of TRIM14 exacerbated hypertrophy and increased phosphorylation of AKT, GSK-3β, mTOR, and p70S6K. This suggested that TRIM14 activated the Akt/GSK-3β/mTOR/p70S6K axis, which in turn promoted the synthesis of hypertrophic factors and fibrotic proteins, contributing to cardiac hypertrophy and dysfunction [52]. Similarly, Chen et al. reported that TRIM27 also drove myocardial hypertrophy by activating the Akt/GSK-3β/mTOR/p70S6K pathway. Mechanistically, TRIM27 interacted with phosphatase tensin homolog (PTEN) to activate the Akt/GSK-3β/mTOR/p70S6K pathway [53]. Yang et al. found that TRIM10 bound to PTEN and promoted its ubiquitination and degradation, which subsequently elevated PIP3 phosphorylation levels and thereby activated Akt, promoting cardiac hypertrophy [54]. In a parallel pathway, TRIM8 was found to be overexpressed in failing human hearts and hypertrophied mouse hearts. Deficiency of this protein alleviated pathological remodeling. Upon stress, TRIM8 translocated to the cytoplasm, ubiquitinated and phosphorylated TAK1, and subsequently activated the pro-hypertrophic P38 and JNK1/2 pathways [55].

Oxidative stress is a well-established driver of hypertrophy, counterbalanced by the antioxidant regulator Nrf2 [56,57]. Han J et al. elucidated a regulatory circuit within this axis: during hypertrophy, upregulation of the deubiquitinase USP28 stabilized TRIM21 via K48-linked deubiquitination. Stabilized TRIM21 then degraded the adapter protein P62, impairing Keap1 sequestration. Consequently, accumulated Keap1 suppressed the Nrf2-dependent antioxidant pathway, exacerbating oxidative stress and hypertrophy [58,59]. Calcium dyshomeostasis represents another core pathological mechanism. TRIM24 disrupted calcium cycling by dual mechanisms: transcriptional modulation of calcium-handling proteins (upregulating RyR2 and CASQ1, downregulating SERCA2a) and impairment of their nanoscale organization. These perturbations accelerated calcium transients, thereby leading to cardiac hypertrophy [60]. Furthermore, ferroptosis, a novel form of regulated cell death, contributes to disease progression. Wu L et al. showed that the deubiquitinase TRIM44 promoted ferroptosis via the TLR4/NOX4 pathway, exacerbating pressure overload-induced cardiac hypertrophy, and fibrosis [61].

#### 3.1.2. TRIMs Inhibit Cardiac Hypertrophy

Several TRIM family members counteract pathological remodeling by attenuating key pro-hypertrophic signaling axes. TRIM32 protected against hypertrophy through multiple mechanisms, including suppression of the Akt/GSK3β/mTOR/p70S6K pathway and promotion of K48-linked ubiquitination and degradation of dysbindin, which inhibited the RhoA-SRF axis [62,63,64]. Intriguingly, its protective effect can be antagonized by TRIM24, which competitively bound to dysbindin and impaired TRIM32-mediated degradation, thereby promoting hypertrophy [62]. In parallel, Liu J et al. showed that TRIM16 alleviated hypertrophy by targeting oxidative stress. It promoted K48-linked ubiquitination and degradation of Src, leading to reduced phosphorylation of the antioxidant protein Prdx1. This enhanced Nrf2 activity, attenuated oxidative stress, and ultimately inhibited pathological cardiac remodeling [65]. Other TRIM proteins function through distinct pathways. TRIM65 conferred protection by interacting with the Jak1/signal transducer and activator of transcription 1 (Stat1) axis. It promoted K48-linked ubiquitination of Jak1, suppressing the Jak1/Stat1 signaling pathway, inhibiting mitochondrial-related apoptosis and autophagy, and thereby alleviating hypertrophy induced by stress [66]. Contrary to TRIM8, TRIM38 exerted its anti-hypertrophic effect by negatively regulating the TAK1-dependent MAPK cascade, inhibiting TAK1 phosphorylation and subsequent JNK/P38 activation, thereby protecting against pathological cardiac remodeling [67]. Arya R et al. found that TRIM63 inhibited activity of PKCε and its translocation to focal adhesions, resulting in the inhibition of FAK. ERK1/2, the downstream effector of FAK, was inhibited, preventing cardiac hypertrophy [68]. Maejima Y et al. revealed that TRIM63 targeted calcineurin A (CnA) for proteasomal degradation by polyubiquitinating it, thereby inhibiting the calcineurin A-nuclear factor of activated T-cell (CnA-NFAT) signaling cascade responsible for cardiac hypertrophy [69].

#### 3.1.3. Conclusions and Perspective

In summary, TRIM family proteins form a complex and mutually balanced regulatory network in cardiac hypertrophy. Certain members, such as TRIM14, TRIM10, TRIM27, and TRIM8, directly drive disease progression by activating pro-hypertrophic pathways like Akt/mTOR, disrupting redox and calcium homeostasis, or inducing ferroptosis. In contrast, some TRIM proteins, such as TRIM32, TRIM16, and TRIM65, can protect the heart by inhibiting the same or related pathways and by enhancing the cellular defense systems. Moreover, some TRIM proteins directly work against each other. For example, TRIM24 blocks TRIM32, further highlighting the dynamic complexity inherent in their regulatory mechanisms. Taken together, these findings not only clarify the important roles of TRIM proteins in cardiac hypertrophy but also provide a key molecular base and possible targets for making precise treatments aimed specifically at the TRIM protein family.

### 3.2. TRIM Proteins in Cardiac Ischemia–Reperfusion Injury

**Figure 4:**
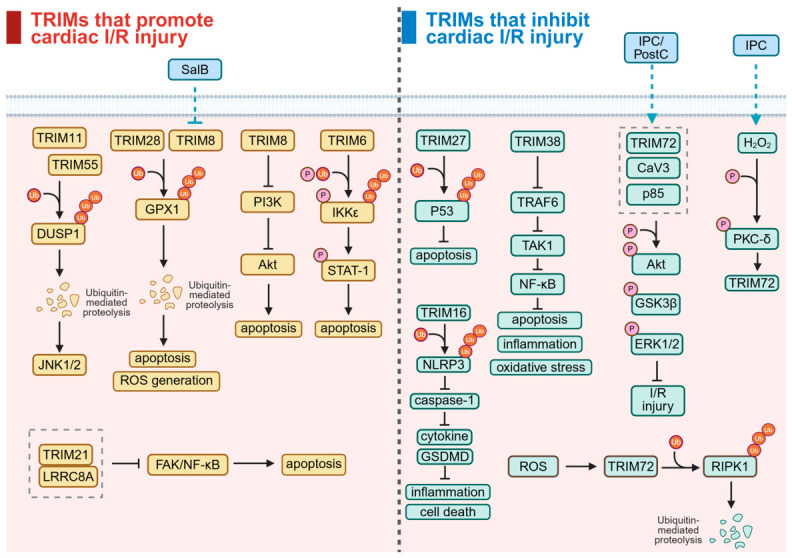
**Dual roles and signaling pathways of TRIM proteins in cardiac I/R injury.** TRIM proteins exert both detrimental and protective effects on cardiac I/R injury through distinct signaling mechanisms. TRIMs that promote cardiac I/R injury are shown in yellow (**left panel**) and TRIMs that inhibit cardiac I/R injury are shown in blue (**right panel**). Left panel shows that TRIM11, TRIM55, TRIM8, TRIM28, TRIM6, and TRIM21 promote I/R injury by enhancing apoptosis, ROS generation, and inflammation via ubiquitin-mediated proteolysis of DUSP1 and GPX1, inhibition of PI3K/Akt pathway, activation of IKKε/STAT-1 pathway and inhibition of FAK/NF-κB signaling. Right panel shows that TRIM27, TRIM38, TRIM16 and TRIM72 protect against I/R injury by inhibiting apoptosis, inflammation, and oxidative stress through p53 ubiquitination, TRAF6/TAK1/NF-κB suppression, NLRP3 inflammasome regulation, modulation of Akt/GSK3β/ERK1/2 signaling and RIPK1 ubiquitination.

The ischemia–reperfusion (I/R) injury refers to the aggravation of ischemic tissue damage following the restoration of blood flow after ischemia. Key mechanisms involve dysregulated pH, oxidative stress, and calcium overload [70]. Cardiac I/R injury often followed the treatment of acute myocardial infarction and led to further cardiomyocyte damage, leading to inhibited treatment efficacy and increased mortality [71]. Although numerous phase II clinical trials, such as those investigating Cyclosporine A, have been conducted over the past decades, no clinical therapy has yet been consistently effective, highlighting the need for novel therapeutic targets [72]. Recently, some TRIM family proteins have been found to be linked with cardiac I/R injury (Figure 4).

#### 3.2.1. TRIMs Promote Cardiac I/R Injury

TRIM11 was found to be upregulated in cardiomyocytes during myocardial I/R to aggravate injury. He F et al. demonstrated that TRIM11 overexpression promoted cardiomyocyte apoptosis by ubiquitinating DUSP1 and then promoting DUSP1 degradation, which in turn sustained downstream target JNK1/2 phosphorylation and activation [73]. Similarly, the pro-apoptotic effect of overexpressed TRIM55 in I/R injured cardiomyocytes was correlated to DUSP1/JNK signaling. TRIM55 decreased DUSP1 protein expression via ubiquitination, which enhanced I/R-induced JNK1/2 activation and cell apoptosis. Furthermore, the microRNA miR-378a-3p was found to be upstream of TRIM55, which inhibited TRIM55 expression, alleviating cardiac I/R injury via TRIM55/DUSP1/JNK signaling [74]. TRIM8 was also found to be upregulated in cardiomyocytes exposed to hypoxic reoxygenation (H/R) treatment, an ideal model for studying cardiac I/R injury at the cellular level. Dang X et al. demonstrated that TRIM8 promoted oxidative stress by increasing ROS production and stimulated apoptosis by inhibiting PI3K/Akt pathway, intensifying I/R injury [75]. Lu B et al. demonstrated that TRIM8 expression was increased in cardiac I/R injury and TRIM8 mediated degradation of the antioxidant enzyme GPX1 via ubiquitination, causing cardiomyocyte apoptosis and ROS generation. Moreover, they also found that Salvianolic acid B (SalB) activity against I/R-induced apoptosis and oxidative stress was partly mediated by inhibiting TRIM8 expression [76]. Taken together, these results indicate that TRIM8 upregulation could promote I/R injury. TRIM6 aggravated cardiac I/R injury by increasing cardiomyocyte apoptosis. Mechanistically, upregulated TRIM6 in myocardial I/R hearts activated IKKε via K48-linked polyubiquitination, which in turn promoted the phosphorylation of Stat1, finally promoting myocardial apoptosis [77]. Hou L et al. found that upregulated Leucine-rich repeat-containing protein 8A (LRRC8A) in the I/R-injured myocardium suppressed the focal adhesion kinase (FAK)/NF-κB pathway by interacting with TRIM21, causing the promotion of cardiomyocyte apoptosis and then the exacerbation of cardiac I/R injury. However, the mechanism by which LRRC8A interacts with TRIM21 remains to be further studied [78]. TRIM28 was significantly elevated in H/R-injured cardiomyocytes and aggravated cardiac I/R injury. Specifically, TRIM28 promoted ROS production, inducing apoptosis by interacting with and negatively regulating GPX1 via ubiquitination [79].

#### 3.2.2. TRIMs Inhibit Cardiac I/R Injury

Li Y et al. found that TRIM27 was significantly reduced in murine hearts following I/R injury and in cardiomyocytes with H/R treatment. They also revealed that TRIM27 protected heart from I/R injury by promoting the k48-linked ubiquitination of p53 to inhibit apoptosis and inflammation of cardiomyocytes [80]. Lu Z et al. found that TRIM38 was strongly decreased in mice with H/R injury, and TRIM38 protected cardiomyocytes from H/R injury by catalyzing the polyubiquitination and degradation of TNF receptor-associated factor 6 (TRAF6) to inhibit TRAF6/TAK1/NF-κB pathway, reducing inflammatory responses, oxidative stress and apoptosis [81]. TRIM16 was found to be downregulated in the heart during cardiac I/R process. Shi M et al. demonstrated that TRIM16 attenuated myocardial I/R injury by directly interacting with NLRP3 and stimulating its K48-linked ubiquitination, contributing to nucleotide-binding oligomerization domain-like receptor family pyrin domain containing 3 (NLRP3) inflammasome inactivation. Interestingly, inflammasome inactivation suppressed caspase-1 activation, which in turn inhibited pro-inflammatory cytokines maturation and prevented Gasdermin D (GSDMD) activation, respectively, causing decreased inflammation and attenuated pyroptosis-triggered lytic cell death [82].

The expression of TRIM72 is reduced during cardiac I/R injury in mice, and its expression restoration under ischemic preconditioning (IPC) and postconditioning (PostC) conditions contributed to the protection of the heart against future lethal I/R injury. Mechanistically, both IPC and PostC restored TRIM72 levels, enabling it to form a functional complex with caveolin-3 (CaV3) and the p85 subunit of phosphoinositide 3-kinase (p85-PI3K). This complex enhanced the phosphorylation of the key pro-survival kinases Akt, GSK3β, and ERK1/2, thereby protecting the heart from I/R injury. In contrast, these protective effects are abolished in TRIM72-knockout mice [83,84]. In recent research, Shan Dan et al. found that repeated short-term IPC increased reactive oxygen species (H_2_O_2_), which induced phosphorylation of PKC-δ at Y311 and then promoted extracellular secretion of TRIM72, protecting hearts from I/R injury [85]. Wang et al. found that TRIM72 could downregulate receptor-interacting protein kinase 1 (RIPK1) expression by proteasome-mediated RIPK1 degradation via K48 polyubiquitination at the sites of K316, K604, and K627 to protect cardiomyocytes from necroptosis and subsequently protect the heart from I/R injury, which was promoted by I/R injury-induced reactive oxygen species (ROS) [86]. Kohr MJ et al. found that IPC and PostC can increase the *S*-nitrosylation (SNO) of the membrane repair protein TRIM72 at cysteine 144 (C144), preventing TRIM72 from H_2_O_2_-induced oxidation and degradation and then reducing cell death mediated by H_2_O_2_. Similarly, the induced mutation of C144 to a serine (TRIM72 C144S) also prevented the irreversible oxidation of TRIM72 at the C144 site, resulting in a reduction in TRIM72 proteasomal degradation and the protection of cardiomyocyte viability [87]. Overall, these findings showed that IPC protected the heart from cardiac I/R injury by blocking TRIM72 breakdown via SNO of TRIM72 or TRIM72 C144S mutation. Additionally, Fillmore N et al. showed that TRIM72 C144S inhibited the release of TRIM72 from cardiomyocytes to protect the heart from I/R injury without impact on cardiac hypertrophy [88]. In summary, TRIM72 inhibited the I/R injury via various mechanisms, promoting the development of novel therapeutic targets.

#### 3.2.3. Conclusions and Perspective

Briefly, TRIM proteins constitute a bidirectional regulatory network in myocardial ischemia–reperfusion (I/R) injury, encompassing both injury-promoting and protective subgroups. This suggests that the fate of cardiomyocytes may depend on the dynamic balance in the expression profiles of different TRIM subgroups during specific pathological stages. Furthermore, although different TRIM proteins act on diverse substrates, their downstream pathways converge significantly, collectively pointing to several core axes of injury, including oxidative stress, inflammatory response, and programmed cell death, such as apoptosis, pyroptosis, and necroptosis. This provides deeper insight into the complex mechanisms underlying I/R injury.

### 3.3. TRIM72 in Atrial Fibrillation

Atrial fibrillation (AF) is a prevalent arrhythmia, affecting about 2% of the general population and 10–12% of those aged 80 and above, with significant risks of stroke, heart failure, and death [89,90,91]. Due to the severe organ toxicity of anti-arrhythmic drugs and the expensive healthcare costs of catheter ablation, new therapeutic strategies are still being studied [92,93]. Atrial fibrosis is recognized as the substrate of atrial fibrillation because it is a sign of structural remodeling [94]. The differentiation of atrial fibroblasts, cellular migration, proliferation, and extracellular matrix synthesis can be activated by the transforming growth factor (TGF)-β1/Smad pathway [95]. Recent studies have focused on the relationship between TRIM72 and the TGF-β1 pathway in atrial fibrosis and atrial fibrillation.

It was found that TRIM72 expression was significantly increased in severe atrial fibrosis and promoted AF by regulating the state of cardiac fibroblasts and the extracellular matrix. Mechanistically, TRIM72 activated the TGF-β1/Smad pathway to regulate myofibroblast differentiation, migration, and proliferation, and extracellular matrix synthesis, stimulating atrial fibrosis and atrial fibrillation [89]. Moreover, Zhang M et al. found that AF patients exhibited an increase in TRIM72 expression while showing a reduction in caveolin-1 (CAV1), an important anti-fibrosis signaling mediator in atrial tissue. An inverse correlation was observed between CAV1 and TRIM72 expression levels, as well as between CAV1 expression levels and the degree of fibrosis in patients with AF. Mechanistically, TRIM72 activated the TGF-β1/SMAD2 signaling pathway to inhibit CAV1 expression, leading to atrial fibrosis and AF [96].

In summary, TRIM72 promotes the progression of atrial fibrillation (AF) through a dual-targeting mechanism, including directly activating classic profibrotic signaling and suppressing the expression of the key anti-fibrotic factor CAV1. This not only suggests a potential plasma or tissue biomarker combination, characterized by elevated TRIM72 and reduced CAV1, but also provides novel therapeutic strategies that shift from single-target inhibition to dual-modal regulatory strategies that simultaneously inhibit TRIM72 function and mimic or stabilize CAV1 activity.

### 3.4. TRIM Proteins in Hypertension and Pulmonary Arterial Hypertension

Systemic hypertension and pulmonary arterial hypertension (PAH) represent two distinct clinical entities that nonetheless share fundamental pathological mechanisms, characterized primarily by maladaptive vascular remodeling, endothelial dysfunction, and chronic inflammation [97,98,99,100]. In both circulations, heightened vascular resistance is driven by the aberrant proliferation of vascular smooth muscle cells (VSMCs) and their phenotypic switching, which shifts the vessel wall from a contractile to a synthetic, proliferative state. Emerging evidence highlights the ubiquitin–proteasome system, particularly the TRIM protein family, as a critical upstream modulator of these pathologies through the regulation of key signaling pathways governing cellular plasticity and oxidative stress [101].

#### 3.4.1. TRIMs Promote Vascular Remodeling and Inflammation

Certain TRIM proteins accelerate the progression of both pulmonary and systemic hypertension by driving excessive proliferation of vascular smooth muscle cells (VSMCs) and intensifying inflammatory cascades. For example, Liu et al. revealed that TRIM59 acts as a central regulator of vascular remodeling in these conditions. In response to mechanical stress and hypoxia, Hippo pathway effectors YAP1 and TEAD4 transcriptionally activate TRIM59, which promotes p53 ubiquitination and proteasomal degradation. Functionally, they revealed that TRIM59 promoted the ubiquitination and proteasomal degradation of p53, thereby removing a critical cell cycle checkpoint and fueling the uncontrolled proliferation of smooth muscle cells, which characterizes medial thickening [102,103].

Similarly, Xu et al. identified TRIM24 as a potent driver of cell proliferation, particularly under hypoxia. Their study showed that TRIM24 significantly enhanced the phosphorylation of AKT at threonine 308. This activation subsequently triggered the mTORC1 signaling axis, evidenced by the increased phosphorylation of downstream effectors S6 and 4E-BP1, which directly drove VSMC hyperplasia and pulmonary vascular remodeling [104].

Regarding inflammatory mechanisms, Chen et al. found that TRIM8 was markedly upregulated by pressure overload. Mechanistically, they observed that TRIM8 catalyzed the K63-linked polyubiquitination of TAK1. This non-degradative modification enhanced TAK1 kinase activity, which subsequently amplified the NF-κB and p38 MAPK signaling pathways, creating a pro-inflammatory microenvironment in the vessel wall [55]. Additionally, Huang X et al. described a malicious positive feedback loop in endothelial cells involving TRIM14. They found that TRIM14 promoted the ubiquitination of specific repressors to sustain endothelial NF-κB activation, thereby perpetuating endothelial dysfunction and vascular inflammation [105].

#### 3.4.2. TRIMs Inhibit Vascular Remodeling and Injury

In contrast to the disease-promoting TRIM proteins, other family members serve as critical endogenous safeguards against vascular damage, shielding against both pulmonary and systemic hypertension. Hu et al. highlighted the protective role of TRIM32, which was significantly downregulated in the pulmonary arteries of PAH patients. They demonstrated that restoring TRIM32 expression effectively inhibited hypoxia-induced proliferation and the migration of smooth muscle cells. Furthermore, they showed that TRIM32 reversed the pathological phenotype switch from a contractile to a synthetic state. Mechanistically, they proved that TRIM32 functioned upstream to dampen the pro-proliferative PI3K/Akt signaling cascade, thereby restoring the expression of key contractile markers such as myocardin and SM22α [106,107].

In the context of systemic hypertension, Zhang et al. identified TRIM31 as a critical suppressor of angiotensin II (Ang II)-induced vascular and renal injury. They observed that TRIM31 expression was suppressed in hypertensive models, and its restoration significantly attenuated blood pressure elevation. Mechanistically, they found that TRIM31 directly interacted with MAP3K7 (TAK1) and promoted its K48-linked polyubiquitination and proteasomal degradation. This targeted degradation blocked the pathological non-canonical TGF-β1 signaling pathway, effectively reducing vascular fibrosis and inflammation [108].

#### 3.4.3. Conclusions and Perspective

The convergence of multiple TRIM proteins on the regulation of vascular smooth muscle cell plasticity suggests the existence of a shared “vascular ubiquitin code” governing both systemic and pulmonary hypertension. A fundamental ubiquitin imbalance underlies disease progression: pathological upregulation of remodeling drivers (e.g., TRIM59, TRIM24) occurs in parallel with the loss of protective guardians (e.g., TRIM31, TRIM32). However, translating these findings requires overcoming critical limitations. Current evidence heavily relies on acute rodent models (e.g., hypoxia or Ang II infusion), which often fail to recapitulate the chronic, multifactorial nature of human vascular remodeling. Furthermore, while systemic and pulmonary hypertension share these molecular mechanisms, they involve entirely distinct hemodynamic environments. Systemic administration of broad TRIM modulators could therefore provoke off-target hemodynamic instability. Future translational efforts must prioritize precision medicine approaches: developing PROTACs to achieve the selective degradation of pathogenic TRIMs or employing gene delivery vectors to restore protective TRIM expression. These strategies aim to reverse maladaptive remodeling while preserving systemic protein homeostasis.

### 3.5. TRIM Proteins in Heart Failure

Heart failure (HF) is a clinical syndrome representing the final stage of many cardiovascular diseases [109,110,111]. While standard pharmacotherapies improve outcomes, they often fail to fully reverse maladaptive remodeling [112,113]. This has fueled interest in myocyte-intrinsic protein quality control systems, particularly the muscle-specific RING finger (MuRF) subgroup of TRIM proteins, which regulate sarcomere stability and stress responses.

#### 3.5.1. TRIMs Promote Heart Failure Progression

Bodine et al. and Willis et al. identified TRIM63 (MuRF1) as the most extensively studied member of the MuRF family, functioning as a pivotal muscle-specific E3 ubiquitin ligase. They established that its consistent enzymatic role was to maintain cardiac protein homeostasis by targeting key sarcomeric and functional proteins—such as myosin heavy chain (MHC), cardiac myosin-binding protein C (cMyBP-C), and titin (TTN)—for proteasome-dependent degradation [68,114,115]. While this baseline activity was fundamentally protective under physiological conditions, Willis et al. observed that experimental overexpression of TRIM63 attenuated hypertrophic responses to pressure overload, whereas genetic deletion exacerbated hypertrophy under similar stress [68,114,115].

**Figure 5:**
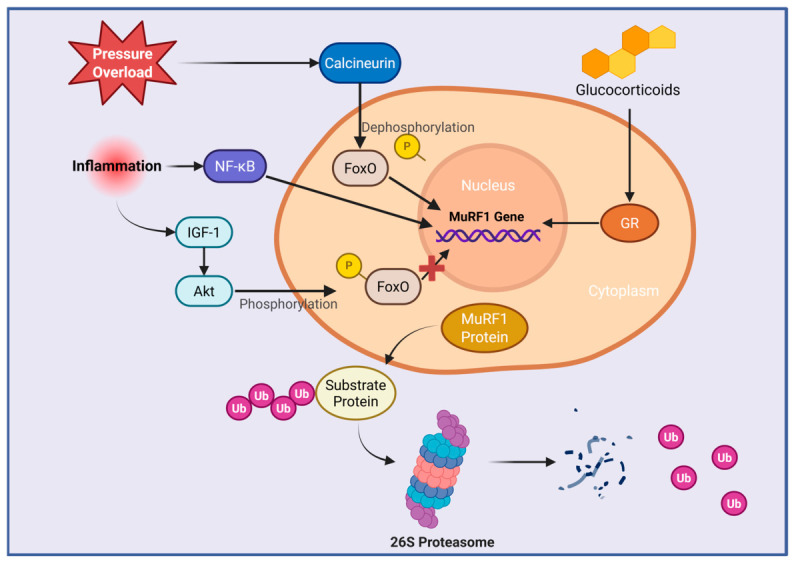
**The regulation of TRIM63 (MuRF1) signaling in cardiomyocytes during heart failure.** Pathological stimuli such as pressure overload and inflammation activate distinct signaling cascades. Pressure overload activates calcineurin, which dephosphorylates the transcription factor FoxO, promoting its nuclear entry and activation of TRIM63 gene expression. Inflammation activates the NF-κB pathway, which also upregulates TRIM63 transcription. In parallel, glucocorticoids can activate the TRIM63 gene via the glucocorticoid receptor (GR). Conversely, the anabolic IGF-1/Akt signaling pathway phosphorylates FoxO, leading to its cytoplasmic sequestration and thereby inhibiting TRIM63 expression. Ultimately, the TRIM63 protein acts as an E3 ubiquitin ligase, targeting proteins for degradation by the 26S proteasome, contributing to cardiac remodeling.

However, in the context of chronic pathological stress, this homeostatic function can become maladaptive. Cai et al. and others revealed that pressure overload, pro-inflammatory cytokines, and activation of catabolic signaling pathways such as the calcineurin–nuclear factor of activated T cells (NFAT) axis and NF-κB led to FoxO-dependent transcriptional activation of TRIM63 [116,117,118,119]. Similarly, Bodine et al. found that glucocorticoids, which were known to induce muscle atrophy, could activate the TRIM63 gene via the glucocorticoid receptor (GR) [114]. In contrast, Cai et al. showed that anabolic IGF-1/Akt signaling phosphorylated FoxO, preventing its nuclear localization and repressing TRIM63 transcription (Figure 5) [116]. Consequently, Kedar et al. demonstrated that excessive TRIM63 activity in advanced HF models drove myocardial atrophy, mitochondrial dysfunction, and contractile impairment, in part through the degradation of essential functional proteins, including cardiac troponin I and creatine kinase [120]. Beyond experimental models, human genetic studies have identified rare variants in TRIM63 as disease modifiers in hypertrophic cardiomyopathy (HCM) [121]. Recent analyses show that specific HCM-associated mutations in TRIM63 directly impair its E3 ubiquitin ligase activity and substrate degradation, confirming that TRIM63-mediated protein quality control is crucial in human cardiac pathology [122].

#### 3.5.2. TRIMs Inhibit Heart Failure Progression

Perera et al. found that TRIM55 (MuRF2) was indispensable for myofibril assembly and alignment during cardiac development and adaptation [123]. They observed that TRIM55 was strategically positioned at the M-line and Z-disc, where it acted as a mechanical strain sensor linking sarcomeric stress to nuclear transcriptional responses that govern protein turnover and structural remodeling [123,124]. Specifically, Lange et al. showed that TRIM55 plays a crucial role in stabilizing the sarcomeric structure [124,125]. Heliste et al. demonstrated that this structural function is essential for maintaining myocardial integrity; they showed that loss-of-function variants (such as E140K) disrupt myofibril organization and lead to heart failure, highlighting TRIM55′s protective role as a structural scaffold [126].

However, this chronic protection contrasts with its pro-apoptotic role during acute I/R injury. Bu et al. indicated that in the ischemic border zone, TRIM55 expression is upregulated and interacts with Nrf2, targeting it for degradation [127]. They further established that by dismantling the Nrf2/HO-1 antioxidant axis, TRIM55 exacerbates oxidative stress and drives cardiomyocyte apoptosis. This functional divergence demonstrates that TRIM55′s role is strictly context-dependent. Under chronic stress, nuclear TRIM55 restrains pro-hypertrophic SRF to preserve myocardial structure. Conversely, during acute ischemia, cytoplasmic TRIM55 degrades DUSP1 to drive apoptosis. This spatial and functional switching highlights its disease-specific complexity.

Regarding TRIM54 (MuRF3), Fielitz et al. reported that it interacted with multiple cytoskeletal proteins, including Four-and-a-half LIM domain protein 2 (FHL2) and β-filamin, targeting them for proteasomal degradation [128]. They demonstrated that loss of TRIM54 resulted in impaired cytoskeletal repair and markedly increased the risk of ventricular wall rupture after myocardial infarction, directly linking TRIM54 deficiency to life-threatening post-infarction mechanical failure [128]. Fielitz et al. showed that TRIM54 and TRIM55 are partially redundant and assemble into hetero-oligomeric complexes to maintain cytoskeletal stability [128]. Consequently, combined deficiency exacerbated structural disarray and rendered the myocardium more susceptible to ischemic and mechanical injury, underscoring their cooperative role as guardians of myofibrillar integrity.

#### 3.5.3. Conclusions and Perspective

The TRIM family’s role in heart failure reveals a complex therapeutic landscape, particularly within the MuRF subfamily. TRIM63 drives pathological atrophy by degrading sarcomeric proteins, while its relatives TRIM55 and TRIM54 maintain structural integrity and mechanical function. Additionally, TRIM55 itself displays disease-specific complexity, protecting against chronic remodeling while exacerbating acute ischemic injury. This functional complexity implies that a blunt therapeutic strategy—such as the indiscriminate inhibition of MuRF E3 ligase activity—could yield deleterious consequences, potentially halting muscle wasting at the cost of compromising myocardial stability and increasing the risk of cardiac rupture. Therefore, the future of TRIM-targeted therapy in heart failure must shift from broad enzymatic blockade to the precision modulation of upstream stress signals. Strategies that specifically dampen the pathological overexpression of TRIM63, perhaps by targeting the FoxO or NF-κB axes, without interfering with the homeostatic housekeeping functions of TRIM55 and TRIM54, represent the most viable path to preserving cardiac function.

### 3.6. TRIM Proteins in Atherosclerosis

Atherosclerosis (AS) is a chronic inflammatory disease and the predominant underlying cause of cardiovascular morbidity and mortality worldwide [129,130]. It is characterized by lipid retention in the arterial intima, endothelial dysfunction, persistent vascular inflammation, and maladaptive remodeling. While lipid-lowering and anti-inflammatory therapies such as statins reduce cardiovascular risk, residual events remain frequent [101,131], indicating a need for novel therapeutic targets. Recent studies identify members of the TRIM family as pivotal modulators of AS pathogenesis, specifically regulating the functions of vascular smooth muscle cells (VSMCs) and endothelial cells (ECs) to either promote or inhibit disease progression.

#### 3.6.1. TRIMs Promote Atherosclerosis

Emerging evidence suggests that specific TRIM proteins accelerate atherosclerosis by driving pathological remodeling in vascular smooth muscle cells (VSMCs) and promoting endothelial dysfunction. In the context of VSMC remodeling, Yao et al. investigated the role of TRIM27 following vascular injury [132]. They found that TRIM27 expression was significantly upregulated and promoted neointimal hyperplasia. Mechanistically, they revealed that TRIM27 interacted with SREBP cleavage-activating protein (SCAP) to form a complex that facilitated the ubiquitination and degradation of IκBα. This degradation event activated the NF-κB signaling pathway, leading to upregulated expression of MMP2 and MMP9, which remodeled the extracellular matrix and promoted VSMC migration [132].

Similarly, Chen et al. identified TRIM5 as a coronary artery disease risk gene. They demonstrated that TRIM5 was transcriptionally activated by Nrf3 under endoplasmic reticulum stress conditions. Their study showed that TRIM5 bound to BECN1 to drive pathological autophagy, which, contrary to its typically protective role, promoted VSMC proliferation, migration, and inflammation in this context, ultimately exacerbating neointimal hyperplasia [133]. Furthermore, Zhou et al. reported that TRIM65 acted as a potent driver of vascular remodeling in VSMCs. They observed that TRIM65 activated the PI3K/Akt/mTOR signaling pathway. Through rescue experiments, they proved that this signaling cascade was responsible for the critical phenotypic switch of VSMCs from a quiescent ‘contractile’ state to a proliferative ‘synthetic’ phenotype, characterized by the marked downregulation of contractile markers such as α-SMA and calponin [134].

In the endothelium, Wang et al. found that TRIM21 played a pro-atherogenic role under disturbed shear stress (DSS) regions. They showed that TRIM21 ubiquitinated and degraded MAPK6. The loss of MAPK6 subsequently enhanced EGR1-mediated NF-κB activation and CXCL12 secretion, which promoted immune cell recruitment and aggravated endothelial inflammation, thereby accelerating lesion formation in ApoE^−/−^ mice [135]. Collectively, these studies highlight that the upregulation of these TRIM proteins drives the core pathological features of plaque progression.

#### 3.6.2. TRIMs Inhibit Atherosclerosis

In contrast to the pro-atherogenic members that drive vascular remodeling, certain TRIM proteins function as critical guardians of vascular integrity by preserving endothelial function and suppressing pathological activation. Ma et al. identified a novel atheroprotective role for TRIM65 specifically within endothelial cells (ECs), which starkly contrasts with its pathogenic role in smooth muscle cells. They demonstrated that TRIM65 expression was upregulated in ECs in response to oxidized low-density lipoprotein (oxLDL). Mechanistically, they found that TRIM65 specifically interacted with Vascular Cell Adhesion Molecule-1 (VCAM-1) and promoted its K48-linked polyubiquitination and subsequent proteasomal degradation. This targeted degradation of VCAM-1 substantially reduced monocyte adhesion to ECs and inhibited the production of inflammatory cytokines, including IL-1β and TNF-α. Furthermore, Ma et al. confirmed the physiological relevance of this pathway in vivo, showing that TRIM65 knockout in ApoE^−/−^ mice resulted in markedly elevated VCAM-1 protein levels within aortic tissues and exacerbated atherosclerotic plaque formation [136].

Additionally, Zeng et al. highlighted the protective function of TRIM59 in maintaining endothelial barrier integrity. They found that TRIM59 bound to Annexin A2, a key regulator of membrane dynamics and cell survival. Through this specific interaction, TRIM59 inhibited endothelial cell apoptosis and significantly reduced the expression of adhesion molecules such as ICAM-1 and VCAM-1. These findings suggest that TRIM59 acts as an endogenous brake on endothelial activation, preventing the initial recruitment of leukocytes to the vessel wall and thereby slowing the initiation of atherosclerotic lesions [137].

#### 3.6.3. Conclusions and Perspective

The involvement of TRIM proteins in atherosclerosis presents a complex, cell-type-specific landscape that complicates therapeutic intervention (Table 1). A compelling example of this dichotomy is TRIM65, which drives disease progression in vascular smooth muscle cells by promoting maladaptive phenotypic switching, yet acts as a protective guardian in endothelial cells by suppressing inflammatory signaling. This cell-type-specific duality suggests that a blunt therapeutic strategy—such as the systemic administration of broad-spectrum TRIM inhibitors—might yield conflicting outcomes or accelerate plaque rupture by exacerbating endothelial injury. Furthermore, current mechanistic insights predominantly rely on murine models (e.g., ApoE^−/−^ mice), which often fail to accurately replicate the complex hemodynamics and advanced instability of human atherosclerotic plaques. Thus, while identifying potent TRIM inhibitors or activators is essential, the future of TRIM-targeted therapies hinges on precision engineering. Strategies such as endothelial-targeted nanoparticles or drug-eluting stents that specifically modulate TRIM activity in distinct vascular cell populations represent the most promising avenue to harness the therapeutic potential of these ubiquitin ligases without triggering off-target vascular damage.

## 4. Therapeutic Prospects and Applications of TRIM in Cardiovascular Disease

This review has shown that TRIM proteins play important roles in both healthy cellular balance and the development of cardiovascular pathology. Furthermore, different TRIM proteins act on diverse substrates to exert their effects, yet their downstream pathways converge significantly, indicating their involvement in complex regulatory networks in cardiovascular diseases. Understanding these regulatory networks is crucial for developing targeted therapies tailored to the specific molecular characteristics of individual patients while avoiding interference with normal metabolic activities. Therefore, exploring and developing novel therapeutic strategies targeting TRIM proteins paves the way for effective cardiovascular disease treatments. In recent years, therapeutic strategies targeting TRIM proteins have mainly focused on a multi-level intervention approach. Based on their mechanisms of action, these therapeutic approaches can be classified into three categories: modulating TRIM expression at the transcriptional or translational level through upstream regulation, interfering with specific TRIM-substrate interactions midstream to prevent subsequent ubiquitination modifications, and intervening downstream in the signaling consequences of TRIM-mediated substrate ubiquitination. Besides these multi-level interventions, emerging strategies are also being extensively studied. First, genetic variant-based therapies identify and use genetic variants in TRIM proteins, which regulate cardiovascular diseases, offering opportunities for precision medicine approaches tailored to target the etiological root. Second, therapeutic prospects based on structural and functional similarities among TRIM proteins recognize both the challenges and opportunities arising from family-wide conservation. By combining interventions at these different regulatory levels along with these emerging strategies, a multi-dimensional therapeutic network can be established, offering significant therapeutic potential for the treatment of cardiovascular diseases.

### 4.1. Upstream Modulation of TRIM Protein Expression

#### 4.1.1. Pharmacological Inducers and Repressors

Direct pharmacological intervention using molecular modulators represents a pivotal strategy for the upstream precise regulation of TRIM protein levels. These agents, which encompass both expression inhibitors and stabilizers, offer precise temporal control over TRIM abundance and function, constituting a challenging and active area of research (Table 2). A recent study found that Nitroxoline (NXQ) can downregulate TRIM25 to increase cell apoptosis by inhibiting sirtuin, a type of histone deacetylase [138], providing a potential direction for AS treatment strategy. Additionally, JP3, a new and non-toxic anti-angiogenic peptide, can phosphorylate TRIM25 to prevent its polyubiquitination-induced degradation and increase its stability, providing the opportunity to develop a JP3 inhibitor to increase TRIM25 levels and then attenuate AS [139]. The mechanism by which the phenolic compound SalB protects cardiomyocytes from I/R injury has also been confirmed to be associated with the downregulation of TRIM8 [76]. Du F et al. revealed that metformin mitigated angiotensin II-induced cardiomyocyte hypertrophy via upregulation of the MuRF1 (TRIM63) and MAFbx pathway [140]. Notably, Chinese herbal medicine exhibits considerable therapeutic potential. Zhang X. et al. found that Zenglv Fumai Granule inhibited TRIM28 expression to attenuate cardiac I/R injury [79].

However, it is unclear how these molecules affect the expression and stability of TRIM proteins, which is to be clarified in further investigations. Furthermore, the undefined dose–response relationship and the toxicity spectrum of these compounds require a systematic investigation.

#### 4.1.2. miRNA- and siRNA-Based Silencing

MicroRNAs (miRNAs) and small interfering RNAs (siRNAs), as two major classes of non-coding RNA molecules, achieve negative regulation of target gene expression through the RNA interference (RNAi) pathway, offering a highly promising strategy for treating cardiovascular diseases by targeting TRIM proteins. miRNAs are endogenous single-stranded RNA molecules, 20–23 nucleotides in length, that regulate protein expression at the post-transcriptional level by mediating mRNA degradation or translational repression through incomplete complementary pairing with the 3′ untranslated region of target mRNAs [141]. SiRNAs are exogenous double-stranded RNA molecules that are processed by the Dicer enzyme into 21–23 nucleotide duplexes. Their guide strand is then incorporated into the RNA-induced silencing complex, leading to the cleavage and degradation of perfectly complementary target mRNAs mediated by Argonaute 2 [142].

In the regulation of TRIM proteins, miRNAs play a crucial negative regulatory role. Studies have shown that various miRNAs can directly target the mRNAs of TRIM family members, modulating their protein expression levels. For instance, in a myocardial hypertrophy model, miRNA-1192 has been confirmed to directly target the mRNAs of TRIM25 and TRIM41, inhibiting their translation. When miRNA-1192 expression is downregulated, the protein levels of TRIM25 and TRIM41 significantly increase, thereby promoting the development of myocardial hypertrophy [143]. MiRNA miR-378a-3p was found to inhibit TRIM55 expression, preventing cardiac I/R injury [74]. This finding reveals that restoring or enhancing the expression of specific miRNAs can achieve negative regulation of pathogenic TRIM proteins, thus exerting therapeutic effects. Unlike the endogenous regulatory mechanism of miRNAs, siRNAs offer a more direct and specific means of gene silencing. By designing siRNAs that are perfectly complementary to the mRNA sequence of a target TRIM protein, efficient degradation of the target mRNA can be induced, achieving precise inhibition of specific TRIM protein expression. In cardiovascular disease research, siRNA technology has been successfully applied to silence various E3 ubiquitin ligases.

Despite the great potential of miRNA/siRNA-based silencing technology for TRIM protein-targeted therapy, its clinical translation still faces numerous challenges. Off-target effects are a major safety concern for RNAi therapies. siRNAs may bind to partially complementary non-target mRNAs, leading to unintended gene silencing and miRNAs, capable of regulating hundreds of target genes, may induce complex network effects due to their pleiotropy, potentially interfering with normal physiological functions [142,144]. Therefore, it is essential to minimize off-target risks of RNAi therapies targeting TRIM proteins through bioinformatic optimization and rigorous validation. Additionally, a targeted delivery system remains a significant hurdle. Current research shows that intravenously administered nanoparticles are primarily sequestered by the liver and spleen, with only a small fraction reaching cardiac tissue. Although vectors such as adeno-associated virus 9 vector enable relatively efficient cardiac transduction, the issue of liver accumulation following systemic administration persists. Moreover, cardiomyocytes, as terminally differentiated cells, have limited capacity to take up exogenous nucleic acids, further complicating delivery. Developing delivery systems modified with cardiac-specific targeting ligands, such as anti-cardiac troponin antibodies, is vital for improving cardiac targeting efficiency [145,146].

#### 4.1.3. Proteolysis Targeting Chimeras

Proteolysis targeting chimeras (PROTACs) are emerging approaches that drive the chemical degradation of a specific disease-related target protein, representing a revolutionary therapeutic strategy that operates at the upstream level to regulate TRIM protein abundance. Structurally, PROTACs are bifunctional hybrid small molecules composed of a target protein ligand (POI ligand) connected via a chemical linker to an E3 ubiquitin ligase ligand (E3 ligand) [147,148]. PROTACs can ensure close spatial proximity between the TRIM protein, which acts as the substrate, and the recruited E3 ligase complex, such as von Hippel–Lindau (VHL) or cereblon (CRBN), thereby hijacking the natural ubiquitin–proteasome system (UPS) to induce selective polyubiquitination and subsequent proteasomal degradation of the TRIM protein [149,150]. This novel protein degradation technology allows for enhanced potency at reduced doses, notably extending pharmacological duration and decreasing the risk of drug resistance [151].

The potential of applying PROTACs in cardiovascular disease has been demonstrated over the past few years. Specifically, Huang et al. developed a bioinspired PROTAC system by encapsulating the degrader (dTRIM24) within PLGA nanoparticles and coating them with an M2 macrophage membrane (MELT). In atherosclerotic mice, this PROTAC system effectively degraded TRIM24 in proinflammatory M1 macrophages because MELT showed enhanced specificity to M1 macrophages, thereby relieving TRIM24-mediated STAT6 acetylation and promoting anti-inflammatory M2 macrophage polarization, ultimately leading to a significant reduction in plaque formation in AS [152].

Despite these promising applications, the development of PROTACs targeting TRIM proteins faces significant challenges. Due to their larger molecular weight, PROTACs often exhibit poor cell membrane permeability and suboptimal pharmacokinetic properties, which can limit their bioavailability and therapeutic efficacy in cardiovascular tissues. Additionally, while most current clinical PROTACs utilize CRBN ligands due to their favorable physicochemical properties, these ligands are prone to cause off-target toxicity through the degradation of neo-substrates. To address these limitations, novel delivery systems such as nanoparticle- or antibody-conjugated PROTACs have been employed for therapeutic benefit [153]. Furthermore, the identification of novel, tissue-specific E3 ligase ligands, including those recognizing TRIM family members themselves, remains a critical unmet need to expand the druggable space and achieve specific protein degradation in tissue [153].

### 4.2. Protein–Protein Interaction Inhibitors

Protein–protein interactions (PPIs) represent a crucial type of therapeutic strategy for cardiovascular diseases by modulating TRIM protein function. PPI inhibitors targeting the RING/B-box/coiled-coil (RBCC) motif domains hold the promise of regulating TRIM protein catalytic activity in a highly efficient and selective manner [154]. This field has made considerable progress over the past decade, successfully developing various peptidomimetics and small-molecule PPI inhibitors for other therapeutic E3 ligase targets. Notably, MDM2 inhibitors [155] and selective ligands for the BIR domains of Inhibitor of apoptosis (IAP) proteins [156] provide important strategic references for developing PPI inhibitors for TRIM proteins.

TRAF6 is functionally analogous to TRIM25, as both are single-polypeptide RING E3 ligases that catalyze K63-linked polyubiquitination in the presence of Ubc13/Uev1A, a modification involved in regulating cell proliferation factor signaling as well as autoimmune and inflammatory responses [157]. The strategy for inhibiting E2/E3 RING interactions has been successfully validated for the protein TRAF6, as well as C25-140, a small-molecule PPI inhibitor of TRAF6/Ubc13-Ub binding was found [158]. In the context of cardiovascular diseases, TRAF6 has been implicated in key pathological processes such as cardiac I/R injury, cardiac inflammation, cardiac hypertrophy, and heart failure. TRAF6-mediated K63-linked ubiquitination can activate signaling pathways of pro-inflammatory cytokines like TNF-α and IL-1β, promoting cardiomyocyte hypertrophy and cardiac fibrosis [159]. Additionally, inhibiting the TRAF6/TAK1/NF-κB pathway can reduce inflammatory responses, oxidative stress, and apoptosis to prevent cardiac I/R injury [81]. Therefore, developing E2/E3 interaction inhibitors for TRIM family members, such as TRIM25, may provide novel strategies for treating myocardial hypertrophy and heart failure.

Furthermore, allosteric regulation is a widespread mechanism in TRIM proteins and RING ligases. It not only provides crucial insights for understanding the regulation of ligase activity but also offers unique strategic entry points for developing novel inhibitors. Studies show that weak intramolecular interactions exist between the coiled-coil domain and the PRYSPRY domain of TRIM25, which is promoted by RNA binding and is essential for the ubiquitination of RIG-I [154,160]. The importance of this regulatory mechanism is validated by the viral protein NS1, which binds to the coiled-coil domain of TRIM25 and inhibits its ubiquitination activity through an allosteric effect [160]. Consequently, the allosteric regulatory mechanisms present in TRIM family members may be key to elucidating and modulating their activities, providing a novel intervention for treating myocardial hypertrophy.

For most RING ligases, dimerization of the RING domain is an essential prerequisite for activation and catalytic activity [161]. This characteristic presents a potential therapeutic strategy to target dimeric TRIM ligases by using PPI modulators to block RING self-association. For instance, the dimerization of TRIM25 is associated with its E3 ligase activity and its regulation of the RIG-I signaling pathway [162]. Therefore, developing small-molecule compounds that can specifically disrupt the dimerization of TRIM proteins holds promise for providing new strategies in cardiovascular disease therapy.

However, the development of PPI inhibitors has long been considered a significant challenge in drug discovery. This is primarily due to the typically large and flat interaction interfaces of protein–protein complexes, which are rich in hydrophobic amino acid residues, making them difficult for traditional small molecules to bind effectively [163]. Furthermore, the peptide nature of many candidate molecules often limits the ability to optimize them into drug-like compounds with favorable absorption, distribution, metabolism, and excretion properties during the drug development process [164]. In the future, integrating multiple strategies to develop TRIM protein PPI inhibitors with high selectivity, favorable drug-like properties, and cardiac targeting potential may cause breakthroughs in the treatment of cardiovascular diseases such as myocardial hypertrophy and heart failure.

### 4.3. Deubiquitinase Regulators

Deubiquitinases (DUBs), a family of proteases capable of reversing ubiquitination modifications, can regulate protein stability, localization, and function by recognizing and cleaving ubiquitin chains on substrate proteins [165]. DUBs and E3 ubiquitin ligases, which include the TRIM protein family, work in concert to maintain the delicate balance of intracellular protein homeostasis. In the field of cardiovascular disease therapy, DUB modulators represent an emerging therapeutic strategy by regulating the stability of both the substrates of TRIM proteins and their downstream targets.

Liu et al. demonstrated that the upregulated USP14, a deubiquitinase associated with the 19S proteasome, promotes the development of myocardial hypertrophy by enhancing GSK-3β phosphorylation. Furthermore, the USP14 inhibitor IU1 significantly suppressed the expression and phosphorylation of the hypertrophy marker β-MHC and reduced GSK-3β, thereby exerting anti-hypertrophic effects [166]. This study suggests that USP14 inhibitors have the potential to be developed as therapeutic agents for myocardial hypertrophy. Cyclindromatosis (CYLD), another DUB, specifically cleaves K63 polyubiquitin chains. Wang H et al. found that CYLD inhibited the ERK and p38/AP1 and c-Myc/Nrf2 pathway to enhance myocardial oxidative stress, thereby promoting cardiac maladaptive remodeling and dysfunction and cardiac hypertrophy [167]. Moreover, CYLD expressions were dramatically elevated in cardiac hypertrophy and heart failure [167], suggesting that CYLD inhibitors may represent novel therapeutic strategies for cardiovascular diseases.

However, insufficient selectivity, poor tissue specificity, and substrate complexity remain major obstacles to the clinical translation of DUB regulators. Future research should focus on developing highly selective DUB modulators, establishing cardiac-specific delivery systems, and conducting rigorous preclinical and clinical validation to advance the clinical translation of this emerging therapeutic strategy.

### 4.4. Genetic Variant-Based Therapies

The discovery of many mutations in TRIM proteins associated with cardiovascular diseases provides a new direction for therapeutic approaches that target the underlying etiology rather than merely alleviating symptoms. Genetic variant-based therapies show greater precision and have significant potential for application in cardiovascular medicine.

Chen SN et al. found that two missense variants, p.A48V (p.Ala48Val) and p.I130M (p.Ile130Met), and a deletion variant, p.Q247* (p.Glu247Ter), in *TRIM63* each caused the loss of E3 ligase activity of TRIM63, promoting cardiac hypertrophy development. Specifically, these rare mutations caused defective auto-ubiquitination, reduced ubiquitination, and proteasomal degradation of known substrates, including myosin heavy chain 6 (MYH6), myosin binding protein C3 (MYBPC3), and protein phosphatase 3 catalytic subunit, beta isoform (PPP3CB). Besides impaired cardiac protein degradation, the mutations activated the mammalian target of rapamycin (MTOR)-S6K and calcineurin-RCAN1.4 pathways, together contributing to cardiac hypertrophy [168]. Moreover, Salazar-Mendiguchía J et al. revealed that *TRIM63* rare variants represented a rare cause of cardiac hypertrophy, with an autosomal recessive pattern of inheritance. Among the identified missense variants and protein-truncating variants of *TRIM63*, p.(Gln247*) was the most common. These mutations reduced TRIM63’s auto-ubiquitination function and substrate ubiquitination while activating the mTOR pathway signaling [169]. Additionally, Su M et al. showed that most rare *TRIM63* and *TRIM55* variants act as modifiers rather than independent causes of cardiac hypertrophy because sarcomere mutations and MuRF variants frequently coexist [121]. It is reported that gene replacement therapy is most effective when applied to pathogenic loss-of-function variants, which is consistent with the findings that *TRIM63* mutations produce nonfunctional proteins. These findings pave the way for the design of genes of interest in the preclinical development of gene replacement therapy for cardiac hypertrophy [170]. An increasing number of TRIM variants linked to cardiovascular diseases have been identified. Hughes MF et al. found that an increased risk of coronary artery disease is associated with the rs11601507 missense variant in *TRIM5*. The variant increased TRIM5 expression level, finally increasing lipolysis and inflammation. A somatic genome editing approach using CRISPR-Cas9 may be effective in removing the *TRIM5* variant because this technology can rectify both loss-of-function and gain-of-function pathogenic variants [170,171]. Because TRIM5 is also a pro-atherogenic factor, the presence of the rs11601507 mutation in AS and the identification of other *TRIM5* variants require further study. Heliste J et al. demonstrated that the rs138811034 variant in *TRIM55* increased the risk of heart failure. This variant upregulated *Nppa*, a gene associated with cardiac stress, and suppressed *Myl7* and *Adra2b*, genes related to cardiac contractility. Moreover, the mutation also reduced cardiomyocyte viability and impaired cell cycle progression [126]. The rs138811034 variant in *TRIM55* can be used as a prognostic marker for heart failure patients and as a therapeutic target. Besides naturally occurring mutations, the induced mutations also have effective potential in treating cardiovascular diseases. For example, TRIM72 C144S can be an effective therapeutic target for inhibiting cardiac I/R injury [88].

However, rare variants need to be validated in other large cohorts to show their accurate prevalence and impact on cardiovascular diseases. Meanwhile, more TRIM gene variants that significantly affect cardiovascular disease remain to be discovered. Moreover, many gene therapies targeting TRIM gene mutation for cardiovascular diseases are still in the preclinical stage, requiring further studies to determine their efficacy and safety. Finally, because transgene expressions in non-target tissues may cause severe side effects, strategies including using tissue-specific promoters or optimizing vector design can be studied to improve treatment precision.

### 4.5. Therapeutic Prospects Based on Structural and Functional Similarities of the TRIM Family

Given that the TRIM family comprises over 80 members sharing conserved structures and participating in diverse biological processes, the therapeutic potential of targeting individual TRIM proteins can be assessed by considering both the challenges and opportunities presented by their inherent complexity.

On one hand, the similarities within the TRIM family cause functional redundancy, suggesting that the overlapping or compensatory functions of diverse TRIM proteins limit the efficacy of inhibiting a single TRIM because other family members may compensate for the role of the inhibited TRIM protein, and structural similarity may raise the risk of off-target effects on related proteins [172,173]. On the other hand, the contribution of different TRIM proteins to the same cardiovascular diseases and the convergence of some downstream signaling pathways of diverse TRIM proteins also facilitate broad-spectrum therapeutic approaches. For example, TRIM21 not only promotes cardiac hypertrophy development but also exacerbates myocardial I/R injury [59,78]. Therefore, inhibiting TRIM21 expression in cardiomyocytes is highly effective for patients suffering from both conditions, as well as for those with one condition, so as to prevent the other, providing new therapeutic direction for cardiovascular disease. Additionally, targeting a common downstream target of TRIM proteins, such as Stat1, can exert multiple effects on different cardiovascular diseases, including the prevention of cardiac hypertrophy and cardiac I/R injury [66,77].

Given the structural and functional similarities between some TRIM proteins, future studies should balance precision strategies that use unique features of individual TRIM proteins, such as tissue-specific expression and distinct C-terminal domains, to address off-target effects with broad-spectrum approaches that exploit TRIM family similarities for multi-disease efficacy. However, broad-spectrum therapeutic strategies are limited by the observation that the same TRIM protein may play opposing roles in different cardiovascular diseases. Therefore, a detailed understanding of the functions of individual TRIM proteins in different cardiovascular diseases and in different cell types is essential. Moreover, further studies are still needed to address functional redundancy.

## 5. Conclusions

**Figure 6:**
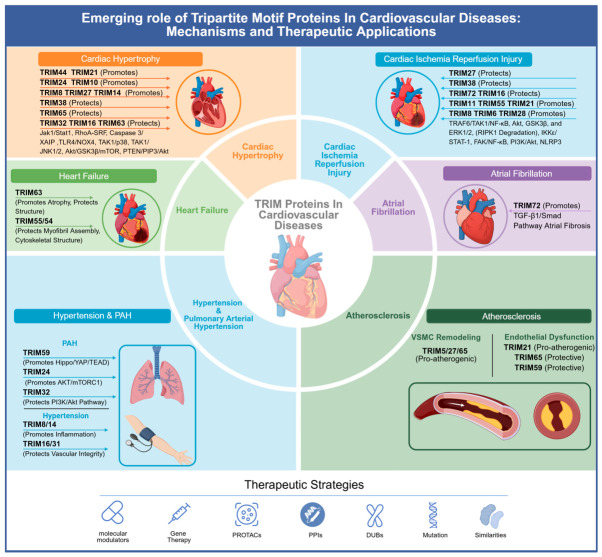
**Overview of TRIM proteins in cardiovascular diseases.** Schematic overview of the emerging roles of tripartite motif (TRIM) proteins in cardiovascular diseases. TRIM family members regulate key pathological processes, including cardiac hypertrophy, ischemia–reperfusion injury, heart failure, atherosclerosis, atrial fibrillation, hypertension, and pulmonary arterial hypertension, either promoting or protecting against disease progression through diverse molecular mechanisms. The central diagram illustrates the dual functional nature of TRIM proteins in cardiovascular pathophysiology, highlighting their potential as therapeutic targets for molecular modulators, gene therapy, PROTACs, PPIs, DUBs, genetic variant-based therapies, and therapies based on structural and functional similarities of the TRIM proteins.

In this review, we summarize the emerging roles of the tripartite motif (TRIM)-containing protein family genes in the progression of cardiovascular diseases by regulating the ubiquitin–proteasome pathway, ferroptosis, calcium homeostasis, autophagy, and other mechanisms, providing novel directions for early diagnosis, treatment, and prognosis (Figure 6). The network of interactions between TRIM proteins and cardiovascular diseases is systemic and interconnected. Based on their E3 ubiquitin ligase activity, the ubiquitination mediated by most TRIM proteins typically involves K48-linked ubiquitination, which leads to proteasomal degradation, or K63-linked ubiquitination, which is involved in cellular signaling through kinase activation. Furthermore, specific ubiquitination sites, such as the sites on RIPK1 mediated by TRIM72, are critical for cardioprotection, and even deubiquitinases lacking E3 activity, like TRIM44, play significant roles in cardiovascular pathology.

Mechanistically, although TRIM proteins target a vast array of specific substrates, their downstream signaling consequences remarkably converge onto several core pathological hubs: excessive reactive oxygen species (ROS) production, persistent inflammatory cascades, and various forms of regulated cell death (such as apoptosis, pyroptosis, and ferroptosis). Ultimately, the exacerbation of these interconnected pathways triggers irreversible cellular damage, particularly the progressive loss of terminally differentiated cardiomyocytes and the severe disruption of vascular endothelial integrity, which serves as the fundamental driver for the deterioration of cardiovascular function.

However, the involvement of TRIM proteins in cardiovascular diseases is highly complex and context-dependent. Since individual TRIM proteins often participate in multiple cardiovascular pathologies, therapeutic strategies must carefully evaluate their systemic impacts. For instance, targeting a shared pathogenic hub like TRIM8 could simultaneously dismantle multiple disease networks. By ubiquitinating TAK1, TRIM8 aggressively amplifies the p38/JNK and NF-κB cascades to drive both cardiac hypertrophy and hypertensive vascular inflammation. In parallel, it exacerbates ischemic injury by degrading the antioxidant GPX1 and suppressing Akt survival signaling. Inhibiting this single multifaceted target thus offers compounded therapeutic benefits for patients with overlapping conditions, such as hypertensive heart disease complicated by ischemia. Conversely, other members exhibit highly context-dependent dualities. Inhibiting TRIM72 may produce opposite effects in AF and cardiac I/R injury. TRIM72 proteins translocate to the membrane damage site to repair the membrane in cardiac I/R injury, while they are present in the cytoplasm of cardiac fibroblasts in AF [87,89]. Moreover, the binding partner of TRIM72 in cardiac I/R injury is CaV3, which is highly expressed in cardiomyocytes; however, CaV1, which is widely expressed in the fibroblasts, is present downstream of TRIM72 in AF [84,174,175]. The post-translational SNO of TRIM72 in the IPC condition may also contribute to the protective role of TRIM72 in cardiac I/R injury [87]. The findings emphasize that the effect of the same TRIM protein on different vascular diseases depends on the cellular location, post-translational modifications, and molecular partners, explaining why targeting TRIM proteins therapeutically requires careful consideration of the specific cardiovascular pathology being treated.

Furthermore, closely related members within the same subfamily, such as the MuRFs in heart failure, can exhibit divergent functions. These complexities indicate that broad-spectrum enzymatic blockade could yield conflicting outcomes or exacerbate underlying dysfunctions. Therefore, the future of TRIM-targeted therapy depends on precise modulation utilizing targeted delivery systems, such as PROTACs, endothelial-targeted nanoparticles, or drug-eluting stents, to restrict TRIM modulation to specific cell populations.

Despite significant recent advances, several critical limitations and challenges must be addressed before clinical translation can be achieved. First, our current understanding relies heavily on murine models and in vitro experiments, which may not fully recapitulate the complexity of human cardiovascular pathophysiology. Second, because the ubiquitin–proteasome system is fundamentally essential for global protein homeostasis, systemic pharmacological intervention carries a substantial risk of off-target toxicity. Third, the structural characterization and precise physiological functions of numerous TRIM family members remain entirely elusive. Furthermore, there is likely complex crosstalk among functional pathways mediated by TRIM proteins, such as overlapping regulatory networks between autophagy and ferroptosis. A more detailed investigation into the cooperative, synergistic, or antagonistic roles of unidentified TRIM family members that may form heterodimers with known TRIM proteins will be crucial for improving therapeutic efficacy. Additionally, exploring the potential of circulating TRIM proteins as non-invasive diagnostic or prognostic biomarkers could offer immediate clinical value. Ultimately, a comprehensive understanding of the TRIM protein system will facilitate the development of precision therapeutics against cardiovascular diseases and offer novel insights for subsequent scientific inquiry.

## Figures and Tables

**Table 1:** The role and mechanisms of TRIM proteins in atherosclerosis (AS).

Cell Type	TRIM	Role in AS	Function(s)	Mechanism	Ref.
VSMCs	TRIM5	Pro-atherogenic	Promote VSMC proliferation and migration	Drives pathological autophagy via BECN1	[133]
	TRIM27	Pro-atherogenic	Promote VSMC migration	Activates NF-κB via SCAP/IκBα pathway	[132]
	TRIM65	Pro-atherogenic	Promote phenotypic switch and proliferation	Activation of PI3K/Akt/mTOR pathway	[134]
Endothelial Cells	TRIM59	Atheroprotective	Maintain endothelial integrity	Binds Annexin A2 to inhibit apoptosis	[137]
	TRIM65	Atheroprotective	Reduce monocyte adhesion	K48-linked degradation of VCAM-1	[136]
	TRIM21	Pro-atherogenic	Promote endothelial inflammation	Degradation of MAPK6 to enhance NF-κB	[135]

**Table 2:** Potential therapeutic agents associated with the regulation of TRIM protein-mediated pathways.

Therapeutic Agent	Target TRIM	Therapeutic Mechanism	Disease Context	Ref.
Nitroxoline (NXQ)	TRIM25	Downregulation of TRIM25 via sirtuin (histone deacetylase) inhibition, leading to increased cell apoptosis	Atherosclerosis (AS)	[138]
JP3 inhibitor	TRIM25	Inhibition of JP3-mediated phosphorylation of TRIM25, promoting polyubiquitination-induced degradation of TRIM25 and reducing its stability	Atherosclerosis (AS)	[139]
Salvianolic acid B (SalB)	TRIM8	Downregulation of TRIM8 expression to protect cardiomyocytes from I/R injury	Cardiac I/R injury	[76]
Metformin	TRIM63 (MuRF1)	Upregulation of MuRF1 (TRIM63) and MAFbx pathway to attenuate cardiomyocyte hypertrophy	Cardiac hypertrophy	[140]
Zenglv Fumai Granule	TRIM28	Inhibition of TRIM28 expression to attenuate cardiac I/R injury	Cardiac I/R injury	[79]

## Data Availability

No new data were created or analyzed in this study.

## Abbreviations

The following abbreviations are used in this manuscript:

4E-BP1	Eukaryotic translation initiation factor 4E-binding protein 1
ACE	Angiotensin-converting enzyme
AF	Atrial fibrillation
AKT	Protein kinase B
Ang II	Angiotensin II
AP1	Activator protein 1
AS	Atherosclerosis
ATP	Adenosine triphosphate
BECN1	Beclin 1
BROMO	Bromodomain
CASQ1	Calsequestrin 1
CAV1	Caveolin-1
CaV3	Caveolin-3
c-Myc	Cellular myelocytomatosis oncogene
cMyBP-C	Cardiac myosin-binding protein C
COS	C-terminal subgroup one signature
CRBN	Cereblon
CVDs	Cardiovascular diseases
CXCL12	C-X-C motif chemokine ligand 12
CYLD	Cyclindromatosis
DSS	Disturbed shear stress
DUBs	Deubiquitinases
DUSP1	Dual specificity protein phosphatase 1
ECs	Endothelial cells
EGR1	Early growth response protein 1
ERK1/2	Extracellular signal-regulated kinases 1/2
FAK	Focal adhesion kinase
FHL2	Four-and-a-half LIM domain protein 2
FoxO	Forkhead box O
GPX1	Glutathione peroxidase 1
GR	Glucocorticoid receptor
GSDMD	Gasdermin D
GSK-3β	Glycogen synthase kinase-3 beta
H_2_O_2_	Hydrogen peroxide
HF	Heart failure
H/R	Hypoxic reoxygenation
HCM	Hypertrophic cardiomyopathy
IAP	Inhibitor of apoptosis
ICAM-1	Intercellular Adhesion Molecule 1
IGF-1	Insulin-like growth factor 1
IKKε	Inhibitor of nuclear factor kappa-B kinase subunit epsilon
IL-1β	Interleukin-1 beta
IPC	Ischemic preconditioning
I/R	Ischemia–reperfusion
IU1	USP14 inhibitor IU1
IκBα	Nuclear factor of kappa light polypeptide gene enhancer in B-cells inhibitor, alpha
Jak1	Janus kinase 1
JNK1/2	c-Jun N-terminal kinases 1/2
JP3	Anti-angiogenic peptide JP3
Keap1	Kelch-like ECH-associated protein 1
LRRC8A	Leucine-rich repeat-containing protein 8A
MAFbx	Muscle atrophy F-box
MAP3K7	Mitogen-activated protein kinase kinase kinase 7
MAPK	Mitogen-activated protein kinase
MAPK6	Mitogen-activated protein kinase 6
MATH	Meprin and TRAF homology
MDM2	Mouse double minute 2 homolog
MELT	M2 macrophage membrane biomimetic nanoparticle
MHC	Myosin heavy chain
miRNAs	MicroRNAs
MMP2/9	Matrix metallopeptidase 2/9
mRNA	Messenger RNA
mTOR	Mammalian target of rapamycin
mTORC1	Mammalian target of rapamycin complex 1
MuRF	Muscle-specific RING finger
NFAT	Nuclear factor of activated T cells
NF-κB	Nuclear factor kappa-light-chain-enhancer of activated B cells
NLRP3	Nucleotide-binding oligomerization domain-like receptor family pyrin domain containing 3
NOX4	NADPH oxidase 4
Nrf2	Nuclear factor erythroid 2-related factor 2
Nrf3	Nuclear factor erythroid 2-related factor 3
NS1	Non-structural protein 1
NXQ	Nitroxoline
oxLDL	Oxidized low-density lipoprotein
P38	p38 mitogen-activated protein kinase
p53	Tumor protein p53
P62	Nucleoporin p62/Sequestosome 1
p70S6K	Ribosomal protein S6 kinase beta-1
p85-PI3K	p85 subunit of phosphoinositide 3-kinase
PAH	Pulmonary arterial hypertension
PHD	Plant Homeodomain
PHOTAC	Photochemically targeting chimeras
PI3K	Phosphoinositide 3-kinase
PIP3	Phosphatidylinositol (3,4,5)-trisphosphate
PKCδ	Protein kinase C delta type
PLGA	Poly(lactic-co-glycolic acid)
POI	Protein of interest
PostC	Postconditioning
PPIs	Protein–Protein Interactions
Prdx1	Peroxiredoxin-1
PROTACs	Proteolysis targeting chimeras
PTEN	Phosphatase tensin homolog
PTMs	Post-translational modifications
RBCC	RING, B-box, coiled-coil
RhoA	Ras homolog family member A
RIG-I	Retinoic acid-inducible gene I
RING	Really Interesting New Gene
RIPK1	Receptor-interacting protein kinase 1
RNAi	RNA interference
ROS	Reactive oxygen species
RyR2	Ryanodine receptor 2
S6	Ribosomal protein S6
SalB	Salvianolic acid B
SCAP	SREBP cleavage-activating protein
SERCA2a	Sarco/endoplasmic reticulum Ca2+-ATPase 2a
siRNAs	Small interfering RNAs
SM22α	Smooth muscle protein 22-alpha
SMAD2	Suppressor of mothers against decapentaplegic 2
Src	Proto-oncogene tyrosine-protein kinase Src
SRF	Serum response factor
STAT1	Signal transducer and activator of transcription 1
STAT6	Signal transducer and activator of transcription 6
TAK1	Transforming growth factor-beta-activated kinase 1
TEAD4	TEA domain transcription factor 4
TGF-β1	Transforming growth factor-beta 1
TLR4	Toll-like receptor 4
TNF-α	Tumor necrosis factor alpha
TRAF6	TNF receptor-associated factor 6
TRIM	Tripartite motif
TTN	Titin
Ubc13	Ubiquitin-conjugating enzyme 13
Uev1A	Ubiquitin-conjugating enzyme E2 variant 1
UPS	Ubiquitin–proteasome system
USP14	Ubiquitin specific peptidase 14
USP28	Ubiquitin specific peptidase 28
VCAM-1	Vascular Cell Adhesion Molecule-1
VHL	Von Hippel–Lindau
VSMCs	Vascular smooth muscle cells
YAP1	Yes1 associated transcriptional regulator
α-SMA	Alpha-smooth muscle actin
β-MHC	Beta-myosin heavy chain
